# Technology-mediated teaching and learning process: A conceptual study of educators’ response amidst the Covid-19 pandemic

**DOI:** 10.1007/s10639-021-10527-x

**Published:** 2021-05-18

**Authors:** Kingsley Okoye, Jorge Alfonso Rodriguez-Tort, Jose Escamilla, Samira Hosseini

**Affiliations:** 1 Writing Lab, Institute for Future of Education, Office of the Vice President for Research and Technology Transfer, Tecnologico de Monterrey, CP 64849 Monterrey, Nuevo Leon Mexico; 2 The MOOCs, Alternative Credentials unit, Institute for Future of Education, Office of the Vice President for Research and Technology Transfer, Tecnologico de Monterrey, CP 64849 Monterrey, Nuevo Leon Mexico; 3 Institute for Future of Education, Office of the Vice President for Research and Technology Transfer, Tecnologico de Monterrey, CP 64849 Monterrey, Nuevo Leon Mexico; 4 School of Engineering and Sciences, Tecnologico de Monterrey, Nuevo Leon CP 64849 Monterrey, Mexico

**Keywords:** Covid-19, Educational innovation, Technology-mediated learning, Hybrid model, Distance education, Higher education

## Abstract

The COVID-19 pandemic has disrupted many areas of the human and organizational ventures worldwide. This includes new innovative technologies and strategies being developed by educators to foster the rapid learning-recovery and reinstatement of the stakeholders (e.g., teachers and students). Indeed, the main challenge for educators has been on what appropriate steps should be taken to prevent learning loss for the students; ranging from how to provide efficient learning tools/curriculum that ensures continuity of learning, to provision of methods that incorporate coping mechanisms and acceleration of education in general. For several higher educational institutions (HEIs), technology-mediated education has become an integral part of the modern teaching/learning instruction amidst the Covid-19 pandemic, when digital technologies have consequently become an inevitable and indispensable part of learning. To this effect, this study defines a hybrid educational model (HyFlex + Tec) used to enable virtual and in-person education in the HEIs. Practically, the study utilized data usage report from Massive Open Online Courses (MOOCs) and Emotions and Experience Survey questionnaire in a higher education setting for its experiments. To this end, we applied an Exponential Linear trend model and Forecasting method to determine overall progress and statistics for the learners during the Covid-19 pandemic, and subsequently performed a Text Mining and Univariate Analysis of Variance (ANOVA) to determine effects and significant differences that the teaching–learning experiences for the teachers and students have on their energy (learning motivation) levels. From the results, we note that the hybrid learning model supports continuity of education/learning for teachers and students during the Covid-19 pandemic. The study also discusses its innovative importance for future monitoring (tracking) of learning experiences and emotional well-being for the stakeholders in leu (aftermath) of the Covid-19 pandemic.

## Introduction

The COVID‐19 outbreak caused several higher educational institutions (HEIs) to close the campuses for the staffs, teachers, and students, and in turn, span different contingency plans and programs for teaching–learning, especially virtual or distance/online education (Bao, 2020; Woolliscroft, 2020). For example, Bao (2020) notes that a great number of the resultant educational models, infrastructures, and programs have been developed by different institutions to maintain/sustain nonstop teaching and learning for the teachers and students, who are otherwise referred to as *stakeholders* to the different learning initiatives and the context of this study. Whereas, the systematic review by Viner et al. (2020) notes that the effects of the different institutions' closure due to the Covid-19 pandemic have significantly reduced the uttermost peak and impact of the outbreak, and have shown to be useful contingency or control measures. Nonetheless, we note that the implications of the different institutions’ closure remained an issue that has to be taken into account especially in ensuring the continuity of education (learning) during and after the Covid-19 pandemic. In essence, the challenge for the educators is now largely focused on how to manage and continue an effective delivery of the educational services and curriculum that they offer to not just the stakeholders, but also the education community and market at large.

Along these lines, Woolliscroft (2020) notes that the Covid-19 situation has disrupted every phase of the academic world, which will inadvertently change even after the pandemic subsides. As an example, one of the areas in which the Covid-19 pandemic has made its impact is typically towards virtual learning environments (VLE), or yet, heightening of distance education in the wider spectrum (Reimers et al., 2020). Moreover, Woolliscroft (2020) notes that the conversion of the face-to-face to online learning settings has been an unfathomable disruptor particularly for the educationalists. The implications for the educators is to ensure an effective transition to the different learning management systems (LMS) or online learning platforms that they use to foster the learning processes for the teachers and students. They will need to rapidly innovate, develop, and implement adequate and alternative solutions to fill the void that those plans have and will consequently span (IEEE, 2020b; Kummitha, 2020; Woolliscroft, 2020).

On the one hand, recent studies note some practical explanations on how the different institutions and policymakers have leveraged the techno-and-human-driven approaches in controlling the Covid-19 outbreak (Kummitha, 2020; Reimers et al., 2020), and the reason as to why technologies such as augmented reality (AR) and virtual reality (VR) are becoming fast the next frontier in educational programs and development (IEEE, 2020b; Woolliscroft, 2020). On the other hand, the aforementioned studies collectively show that, although the new and emerging technologies are promising, they will never entirely replace the face-to-face (in-person) encounters that constitutes the educational models and curriculum. Thus, the notion of hybrid educational models such as the HyFlex + Tec described in this study.

The main research questions of this study are as follows:

1. How can we analyze the data usage and survey provided by the students and their teachers to understand what have been their overall teaching–learning progress and emotional well-being amidst the Covid-19 pandemic and contingency plans?
2. How can we utilize the resultant information to understand the influential factors, and how this may differ by students vs teachers considering the energy levels (teaching–learning motivation), and how this can be used to support the decision-making strategies and curriculum development for educators in leu (aftermath) of the Covid-19 pandemic?

Thus, based on the stated research questions and objectives, this work makes the following contributions to knowledge:

1. It provides a conceptual study on prevailing factors that impacts the teaching–learning progression and well-being of the stakeholders (teachers and students) during the Covid-19 pandemic.
2. It defines a hybrid model for teaching and learning in higher educational institutions that proves useful in the tracking, monitoring, continuing, and sustaining of teaching–learning processes for the stakeholders amidst the Covid-19 pandemic.
3. It provides a method which uncovers state-of-the-art in learning process and trends that can be continued into the future following the aftermath (post-Covid) of the pandemic.
4. It demonstrates the benefits of data-structure approach such as the text mining technique and its conceptual application within the educational domain to understand the impact and implications of the stakeholders’ views or perspectives on the teaching and learning process especially during the time of crises such as the pandemic.
5. It provides an empirical discussion on why the technology-mediated education may not be sufficiently implemented in higher educational institutions particularly at a time when the digital or educational technologies has become an inevitable part of teaching and learning instruction and/or curriculum development in the several institutions.

## Background information

COVID-19 threatened the effectiveness of education and could have long-term effects to learning and development both for the educationalists and the education community at large (Rogers & Shwetlena, 2020; Viner et al., 2020). The impact or implications of the Covid-19 pandemic on continuing education have not been overemphasized both in theory and in practice (Burgess & Sievertsen, 2020; Chick et al., 2020; Crawford et al., 2020; Reimers et al., 2020; Rogers & Shwetlena, 2020; UNESCO, 2020). For instance, while Crawford et al. (2020) notes that Covid-19 have created significant challenges for the global higher education community (UNESCO, 2014), existing studies shows that the main impact of the Covid-19 outbreak for educators has been on schools being closed in several countries, resulting in students being out of school worldwide (Rogers & Shwetlena, 2020; Setiawan, 2020; UNESCO, 2020). According to the report by United Nations Educational, Scientific and Cultural Organization (UNESCO), the height of the Covid-19 impact was recorded in mid-April, 2020, with approximately 1.6 billion leaners being affected due to the schools closure in response to the pandemic in the different countries, totaling 90.1% of learners worldwide and 192 affected countries (UNESCO, 2020). Perhaps, those figures were anticipated to continue to increase if adequate measures were not taken by the educationalists.

Indeed, the main challenge for the educators is not only on how to cope with the schools’ closure, but also identify what appropriate steps should be taken to prevent the students’ learning loss (Chick et al., 2020; IDB, 2020; Reimers et al., 2020; Rogers & Shwetlena, 2020; Woolliscroft, 2020). According to Rogers and Shwetlena (2020) those measures include the provision of administrative policies and strategies (Nagel et al., 2020) to help prevent the students from dropping out of school, ensuring that they are learning effectively in healthy conditions, and using new innovative technologies and platforms to nurture the rapid recovering and learning of the stakeholders in preparedness for the aftermath (post-covid) of the pandemic. In turn, the educators are expected to act and adopt a three-step turn around policy that incorporates (i) coping mechanism to (ii) managing continuity of learning, and then (iii) improving and acceleration of education (Rogers & Shwetlena, 2020) through innovative methods such as the HyFlex + Tec model described in this study that embodies the three aforementioned components.

On the one hand, while the use of distance learning programs and open educational applications (AIESAD, 2020; Chick et al., 2020; LALA, 2020; OECD, 2017) have inevitably been the most effective platforms through which the schools and teachers can remotely reach the learners and to limit the educational disruptions (Setiawan, 2020; UNESCO, 2020). On the other hand, Crawford et al. (2020) note that the contingency plans and response by the HEIs have been miscellaneous; ranging from having no response at all to the different lockdown strategies and social isolation on campuses, and then rapid re-design of the curriculum to offer fully-online learning platforms. In any case, Chick et al. (2020) argues that maintaining and ensuring the safety of the stakeholders in these circumstances is also paramount. Although, the study (Chick et al., 2020) note that there are no substitutes for hands-on or face-to-face learning which may be better ways to mitigate the loss of learning experiences and acquaintance for the stakeholders during and after the pandemic. To this end, the authors (Chick et al., 2020) propose the use of several innovative solutions; ranging from flipped classroom models to teleconferencing in place of in-person lectures, online practical questions to procedural simulations and virtual videos that are used to facilitate the learning process. They mention that innovative solutions for the sponsors (e.g., the HEIs) that focus on using the aforementioned technologies could help bridge the educational gap for the stakeholders in question (teachers and students) during this unprecedented time of the Covid-19. Interestingly, Pettersson (2020) harbors the idea of how digitalization is planned for and enacted within educational settings. The author (Pettersson, 2020) argues that, for a broader perspective on the concept of digitalization, schools must embrace or deal with digital and educational change particularly when viewed as a process involving cultural-historical activity theory and transformation.

In the same vein, this study shows that a flexible and digital model that takes advantage of educational technologies to create a hybrid and distance learning experiences for the stakeholders, will absolutely ensure that the students do not lose out on the face-to-face interactions or learning, and that the voids are filled. This can be achieved by ensuring that the teaching–learning processes are adjusted (adapted) to diverse requirements by the stakeholders’ circumstances irrespective of where learning occurs (face-to-face or online).

## Methodology

**Fig. 1:**
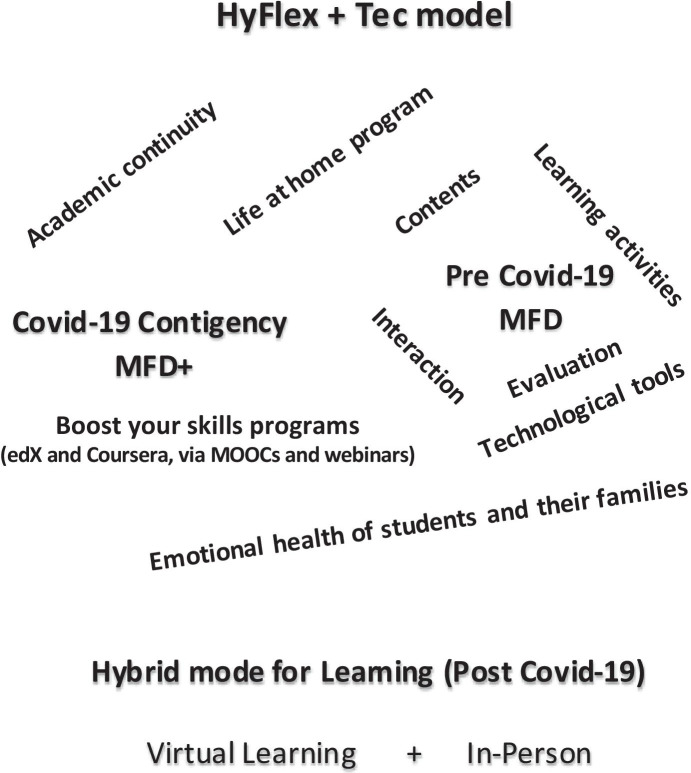
HyFlex + Tec hybrid mode (model) for teaching and learning

Developed in a higher education setting, HyFlex + Tec is a *hybrid* and *innovative* model that allow the students to combine face-to-face and remote learning activities (TEC, 2020c). The model (HyFlex + Tec) is developed by a University in Latin America as part of its initiatives to foster and continue high-quality education and learning experience for the stakeholders following the aftermath of the Covid-19 pandemic. As illustrated in Fig. 1, the model incorporates the learning process for the teachers and students with a flexible digital plus model (MFD +) (TEC, 2020d) that the institution applied during the first few months of the Covid-19 outbreak. The MFD + was developed as an additional framework to the flexible and digital model (MFD) (TEC, 2020a) which was a pre-Covid model for the HEI (see: Fig. 1). This includes different technologies that are methodologically used to support academic continuity for the HEI (e.g., Canvas, Blackboard, etc.), the Emotional health of students and their families, Life at home, and Boost your skills programs to complement learning using platforms such as edX and Coursera (MOOCs), etc.

### Main components of the HyFlex + Tec model

As described in Fig. 1, the HyFlex + Tec model is construed on a three-component design framework or mechanism as follows:

#### Flexible and digital model (MFD) (pre-Covid)

The MFD model incorporates cutting-edge technologies and innovative teaching strategies to facilitate the teaching–learning process for the students. As illustrated in Fig. 1, the MFD model constitutes of (i) Technological tools (ii) Learning activities (iii) Interaction (iv) e-Contents, and (v) Evaluation mechanisms that are collectively designed to ensure the continuity of learning for the students through a flexible digital education approach. The main scope of the MFD model are outlined as follows (TEC, 2020a):

i. *Technological tools*: Use of cutting-edge educational technology to facilitate distance learning experiences for the students.
ii. *Learning activities:* The ease of continuing the teaching of classes through web conferences, active learning sessions, and collaborations.
iii. *Interaction:* Flexibility to conduct online classes from any location.
iv. *e-Contents:* Availability of e-content materials, and resources to support the learning processes (e.g., videos, presentations, infographics, simulations, and web pages, etc.)
v. *Evaluation:* Remote assisted work, advice and feedback, support, and follow-up by the teachers.

#### Flexible digital plus model (MFD +) (Covid-19 contigency)

In response to the Covid-19 outbreak, the HEI implemented new components that complements the MFD model in order to ensure that the training and learning experiences are maximized for the students under the unusual circumstances (TEC, 2020d). As gathered in Fig. 1, the enhanced model (MFD +) constitutes four main additional elements to the MFD as follows:

i. *Academic continuity:* The HEI provided a virtual laboratory (Canvas) that includes an online digital library with a collection of 2.5 million electronic documents (such as books, articles, and videos) and learning/research materials with more than 133 databases. And, a dedicated website to advise and support both the teachers and students in resolving any technical or learning issues they may have in relation to their distance education and learning experience.
ii. *Boost your skills program:* Designed to enable the students to flourish, make the most of their free time, and unleash their potentials by developing new competencies and skills. The HEI made alliance with educational content developers (i.e., Coursera, and edX) to provide the students with unlimited access to specialized programs and certificates from top universities in the world during the months of the Covid-19 outbreak. The students were given opportunity to choose from over 4,000 courses (IFE, 2020a) that allows them to complement their learning, develop soft skills, and earn certificates with similar value as their academic degrees.
iii. *Life at home program:* To complement and continue the students integral education and interaction with other students from different campuses while away from their campus; the HEI established over 1,200 virtual activities and distance learning programs that not only focused on boosting the intellectual capacities of the students, but also were focused on the integral wellbeing of the students. This includes a wide variety of technology-based, at-home sports events, with 50 free MOOCs courses covering different topics on health, music, art, leadership, psychology and well-being.
iv. *Emotional health of students and their families:* In order to protect the students from developing adverse emotions or effects of being isolated and observing the social distancing practices; the HEI created a special platform that provides the students with information and resources to help maintain and strengthen their emotional health. This includes practical contents, tips and recommendations, and personalized activities for balanced emotional health for the students masterminded by degree program directors, advisors, and mentors.

#### HyFlex + Tec hybrid learning mode (post-Covid)

In addition to the MFD and MFD + models described in the earlier sections, the HyFlex + Tec model incorporates the *virtual *plus* in-person* learning components to facilitate the learning processes for students following the aftermath of the Covid-19 pandemic. As gathered in Fig. 1, the Virtual plus In-Person mode of learning is designed to deliver quality education for the students in a hybrid manner by amalgamating the key elements of the MFD and MFD + as follows (TEC, 2020a):

- *Flexibility:* ensuring that teaching and learning processes are adjusted/adapted to diverse requirements by the teachers and students circumstances irrespective of where learning occurs (face-to-face or online) or time factors.
- *Digital:* taking advantage of educational technologies (EdTech) to create a hybrid and distance learning experiences for the stakeholders (teachers and students).

Theoretically, the HyFlex + Tec model (Fig. 1) is an innovation from academic experts that comprises of the cumulative experiences of the HEI in distance education and international experiences over the years (TEC, 2020c). It involves schemes and developments that have emerged through feedbacks and consultations with the stakeholders (teachers, students, academic employees, parents, and educational organizations) with the aim of attaining effectiveness in the programs. This includes exchange of knowledge, experiences, and advice from over 150 universities, organizations such as the Columbus Association of European and Latin American Universities, and Council for Advancement and Support of Education (CASE) (TEC, 2020a). Moreover, the main objective of the model or scheme is to ensure that learning and academic training continues with the highest quality standards in the buildup and aftermath of Covid-19 and attaining a safe learning environment (Bao, 2020; TEC, 2020c; Viner et al., 2020).

Fundamentally, in order to ensure that the students do not lose out on the face-to-face interactions/experiences or that the voids are filled; the hybrid educational model such as the HyFlex + Tec described in this study was deemed to be paramount. For instance, we note that a substantial increase in the participation and learning outcome of the students in the MOOCs programs could be a good indicator of the effectiveness and positive outcome of the boost your skill element of the HyFlex + Tec model designed to enable the students to flourish, make the most of their free time during the pandemic, and unleash their potentials by developing new competencies and specialized programs (Torres-Barreto et al., 2020), and obtain certificates from top universities in the world. Moreover, in the buildup of the Covid-19 pandemic, the Flexible and Digital model implementation was targeted to facilitate distance education for over 90,000 students, 10,000 teachers, and 55,000 online sessions each week. Prior to the start of the MFD model, 9,923 teachers were trained with over 11,000 h of training. The deployment of the model had presence in the 26 campuses of the institution nationwide. Recently, the report between Feb-June (TEC, 2020a) shows that the institution have invested over 82,483 h in the monitoring of the teaching of the model, with over 135,828 online sessions facilitated by the teachers covering over 3,480,568 students during this period, which in the same vein, may explain the learning progression of the students as observed in the results of the data analysis in this study. More importantly this shows the implication of the innovative models and students' choices under the current circumstances and formative experiences for the educators in terms of future learning management and strategies (Nagel et al., 2020; Tóth & Surman, 2019). Besides, we note from the MFD Experience and Emotion Survey analysis that only 18% of the students required extra support with their learning and well-being during this period.

### Data sampling

This study makes use of two sets of data from the Massive Open Online Courses (MOOCs) (IFE, 2020b) and MFD Emotions and Experience Questionnaire (TEC, 2020b) from the host institution to carry out the experimentations and analysis in this paper. While the MOOCs data consist of the data usage report for the Coursera and edX program’ offered by the HEI between January to June 2020 covering the pre-Covid and Covid-19 era. The MFD Emotions and Experience Questionnaire is a survey designed by the HEI and applied on a weekly basis for collection of information about the well-being and experiences of the students and their teachers in respect to the Flexible Digital Model (TEC, 2020a) during the Covid-19 contingency. Essentially, the work utilized the survey data collected over a period of 8 weeks for the students and 12 weeks for the teachers, respectively, to conduct the analysis presented in this paper. Considering the data sample, we note that while the MOOCs dataset contains information about the different programs, enrollment information, estimated learning hours, course grades and overall progress by the students. The MFD survey data contains information about the teachers’ and students’ distribution, emotional valence and experiences, overall emotions and energy levels during the Covid-19 contingency. From an ethical point of view, the teachers and students who have completed the survey were informed about the purpose for which the questionnaire was being administered, but were not directly involved in the analysis of this study, and their personal information remained anonymous. Likewise, for the MOOCs (Coursera and edX) data, although the learners were not directly informed about the use of the data usage report from the platform, the institutional representatives were fully involved in the expert and ethical discussion of the data to protect the students’ identity and anonymity, and in turn, support the use of the analytic data from the courses.

Statistically, the MOOCs data used for the analysis consist of a total of *n* = 11,691 (edX) and *n* = 66,859 (Coursera) samples with cumulative usage count of 17,574,087 edX and 1,603,893 Coursera online users recorded for the different offered programs between January to June, respectively. For the MFD survey data, we note an initial sample of *n* = 4856 responses for the students and *n* = 946 responses for teachers. After cleaning out the dataset by removing the incomplete variables considered for the study, we note a total sample of *n* = 3869 for students and *n* = 925 for teachers used throughout the analysis in this paper.

#### Research instrument and statistics

The MOOCs (Cousera and edX) data usage report and MFD Emotions survey items went through several stages of validation in order to ensure the reliability and validity of the collected data. A focus group discussion by a group of experts within the educational innovation research and the institutional representatives in charge of the MOOCs program, was carried out to have a clear understanding of the connotations and evaluation mechanisms of the different items or variables we considered for this study (Brown, 2019). The estimated minimum sample size for each of the two datasets was 40 records which we assumed to be the scientifically acceptable sample size (*n* > 30 or 40) (Roscoe, 1975) for conducting the analysis and procedures in this study when compared to the larger enough sample size we have used (see: Data Sampling).

For the survey, the questionnaire which the participants answered were made up of both multiple choice, single choice, and ranked Likert-scale questions. Thus, given that the questionnaire and items we have considered for this study were a combination of the multiple choice, single choice, and ranked Likert-scale questions with varying scales of measurement, we applied a factorial analysis (Cortina, 1993; Green et al., 1977; Jasper, 2010; Tate, 2003) to test the reliability and validation of the data and results of the analysis done in this paper (see: Table 1). The Principal Components Factor Analysis (PCA) with Varimax Rotation (Allen,^2017^; Brown, 2019) was used to analyze the different items in the MOOCs and MFD Survey data to determine its reliability and adequacy in answering the research questions and objectives as reported in Table 1.

**Table 1:** Principal Components Factor Analysis (PCA) with Varimax Rotation for the two datasets (MOOCs and MFD Emotion Survey)

Research Instrument and Item Statistics				
Dataset	Item	Mean	Std. D	Scale
Students MFD Survey	student_category	1.93	0.726	Ranked Likert
	energy_levels	2.35	0.825	Ranked Likert
	emotional_valence	-1.28	0.666	Ranked Likert
	help_needed	0.27	0.446	Single-choice Rank
	overall_emotion	11.77	6.047	Single-choice Rank
Teachers MFD Survey	energy_levels	3.31	0.856	Ranked Likert
	experience_ratings	3.95	0.863	Ranked Likert
	overall_emotion	13.75	6.257	Single-choice Rank
	teacher_category_int	2.08	0.582	Ranked Likert
Coursera	Course Grade	21.50	35.65	Continuos
	Estimated Learning Hours	3.28	6.82	Continuos
	Overall Progress	26.20	36.01	Continuos
	Enrollment period (Month)	3.50	2.15	Ordinal
edX	Cumulative count	2486.89	2471.99	Continuos
	Month of Enrollment	3.55	1.76	Ordinal
Reliability Statistics:	KMO	Bartlett’s Test	(Sig.)	
Students MFD Survey	0.563	643.53	0.000*	
Teachers MFD Survey	0.565	369.53	0.000*	
Coursera	0.669	156,144.76	0.000*	
edX	0.500	125.22	0.000*	

Significance level (*p* <  = 0.05)

The results of the PCA analysis (Table 1) shows that the different items we have used for the study were valid and reliable (adequate) for answering the research questions and objectives, with Reliability statistics outlined as follows; Students MFD Survey (KMO = 0.563, Bartlett's Test = 643.53, p = 0.000), Teachers MFD Survey (KMO = 0.565, Bartlett's Test = 369.53, p = 0.000), Coursera (KMO = 0.669, Bartlett's Test = 156,144.76, p = 0.000), edX (KMO = 0.500, Bartlett's Test = 125.22, p = 0.000), where Eigenvalue > 1, KMO = Kaiser–Meyer–Olkin Measure of Sampling Adequacy (Ermatita et al., 2019; Frost, 2020; Goni et al., 2020; Sevincer et al., 2017).

### Experimental setup

The experimentations in this study were carried out to determine:

- Statistically, the overall progress for the learners (edX and Coursera) during the Covid-19 pandemic.
- The learning experiences and emotional well-being (MFD survey) of the students and teachers during the Covid-19 pandemic.
- The impact that the experiences, emotional valence, and overall emotions have on the energy levels of the stakeholders during the Covid-19 taking into account the different categories of the students and teachers.
- The influence that the aforenoted factors/variables and results have on the students who have indicated if they needed help or not amidst the Covid-19 pandemic.

### Data analysis and results

For the data analysis, we performed an “Exponential linear trend model and forecasting indicator analysis” to determine the extent or proportion of overall progress for the students using the MOOCs data. Also, we applied a “text mining technique” that includes a WordCloud or frequency of terms, and Emotional valence analysis (sentiment analysis) to determine the impact (intensities) of the terms or emotions expressed by the students and their teachers in the survey. Finally, we conducted a “Univariate analysis of variance” (ANOVA) to determine the marginal mean effects that the experiences, emotional valence, and overall emotions expressed by the participants in the survey, have on the energy levels (motivation) of the students and their teachers. This includes a further univariate analysis to determine the potential cause factors for the students who have indicated if they needed help or not. These are presented in detail in the subsequent sections of this paper.

#### Learning outcomes and progress of students

**Fig. 2:**
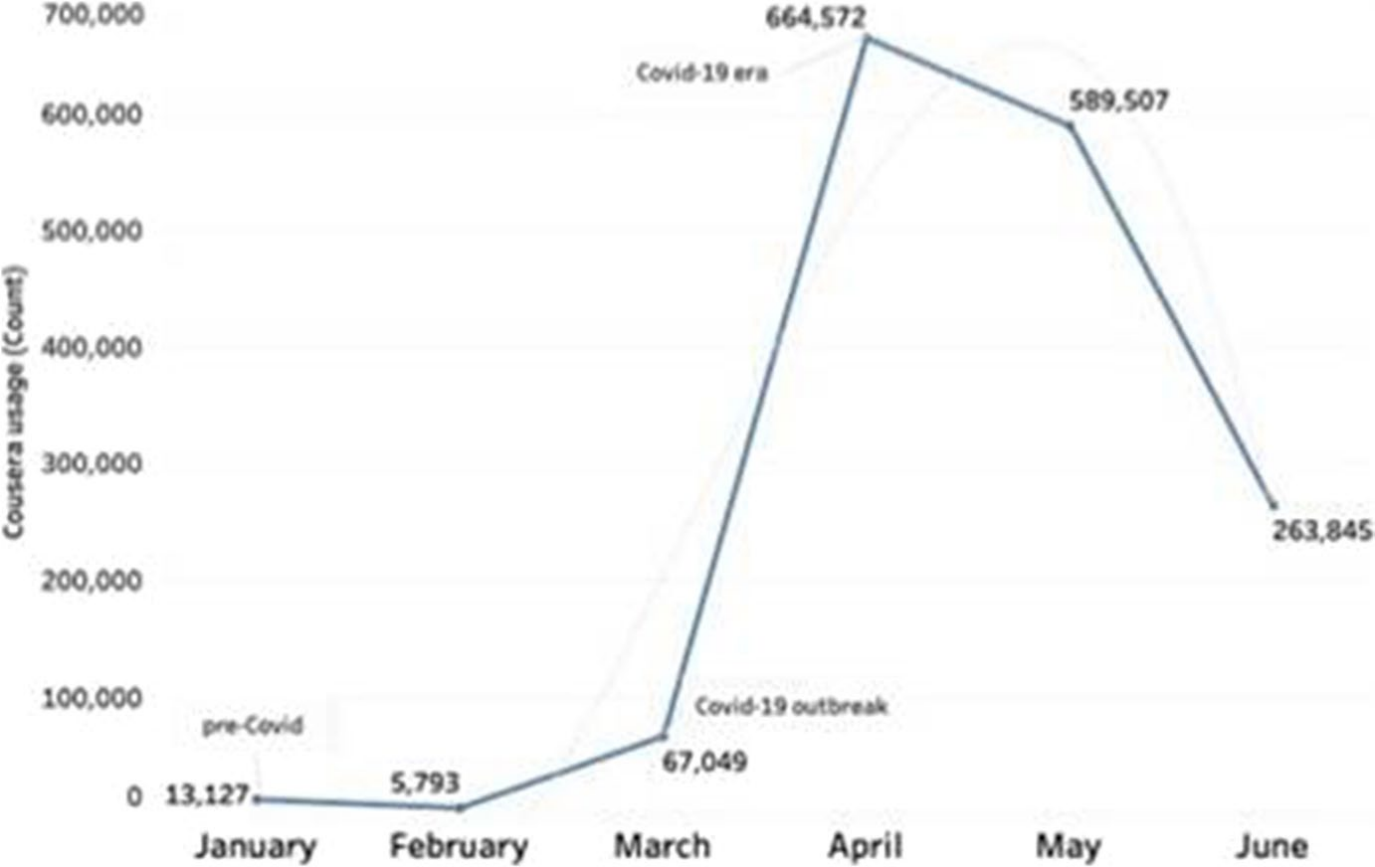
Coursera online learning and usage during pre-Covid and Covid-19 era

**Fig. 3:**
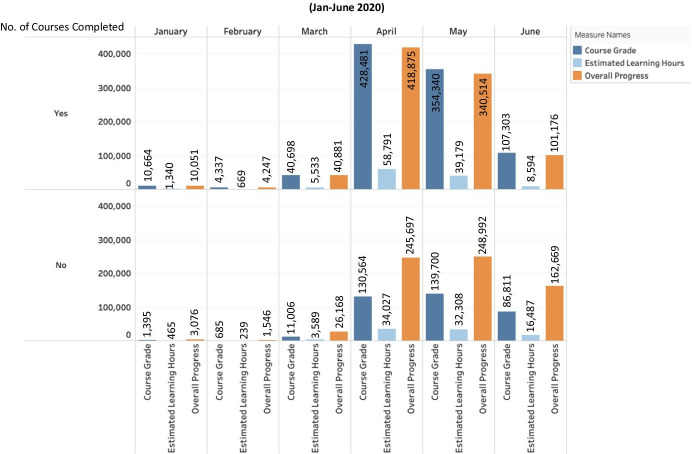
Course grade, estimated learning hours, and overall progress for learners

The study analyzed the dataset from the MOOCs (edX and Coursera) to determine the overall progress and statistics for the learners during the Covid-19 contingency. It used the Tableau Business Intelligence and Analytics Software (Tableau, 2020) to explore the trend and patterns in the data usage report for the edX and Coursera programs. As shown in Figs. 2 and 3, results of the analysis shows that there has been a great increase in the number of learners that are enrolled in the different edX and Coursera programs offered across the HEI.

In Fig. 2, the significant shift or trend for the Coursera programs was observed in the buildup from March when the pandemic began with April (41.43%) and May (36.75%) making up the majority (80.18%) of the transition across the data, respectively (see: Fig. 2).

Furthermore, in Fig. 3, we note that not only have the overall progress for the learners improved during the Covid-19 (March-June), but when we analyzed the performances (e.g., Course grades) for the students, we note a significant increase in the grades in proportion to the learning progress (see: Fig. 3). This was observed for both learners that have completed the different online programs and the ones that were still undergoing the various courses, respectively (Fig. 3).

**Fig. 4:**
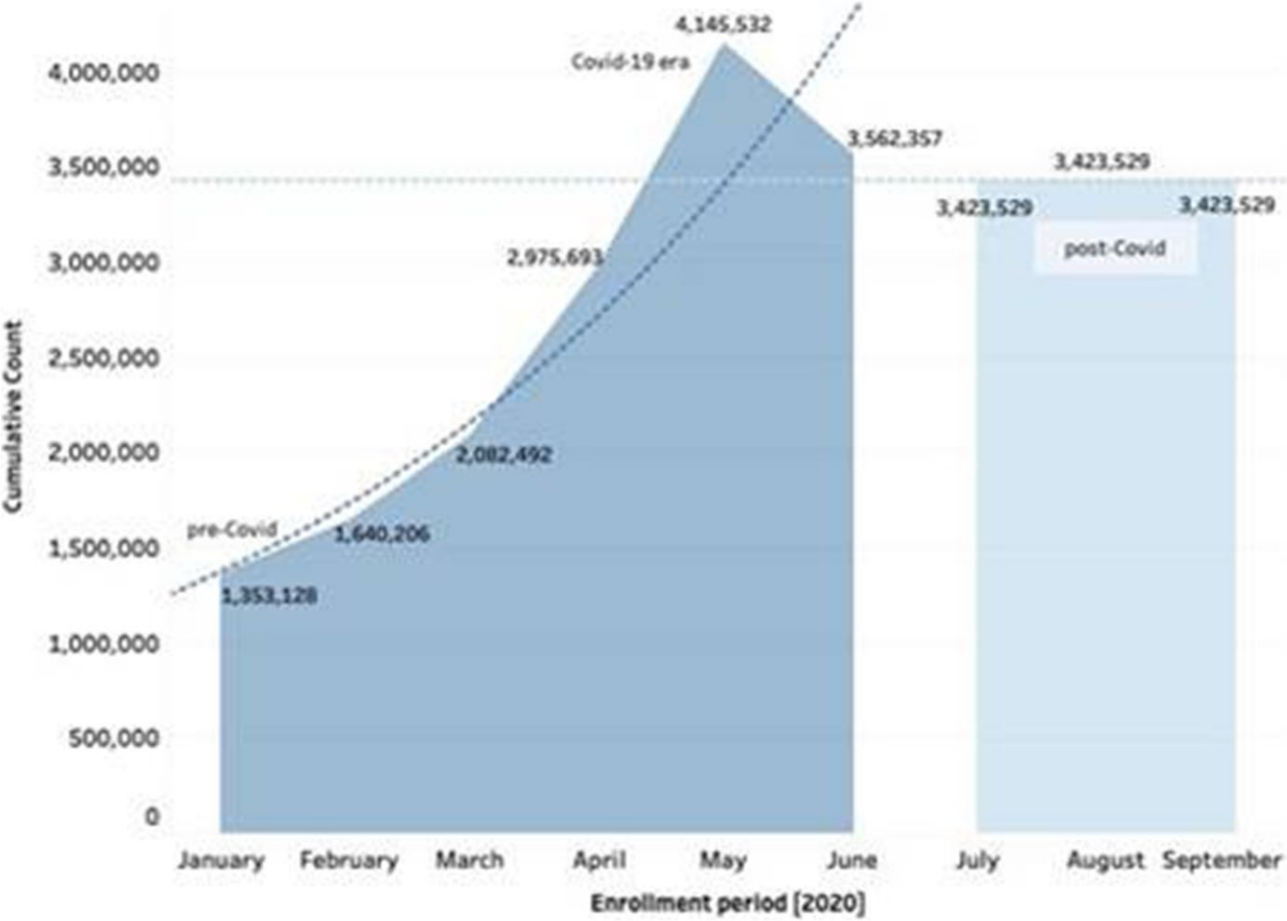
edX online course usage and forecasting (pre-Covid and post-Covid)

To highlight the learning progression or trends that could be continued into the future across the pandemic period (pre-Covid–Covid-19–post-Covid), in Fig. 4 and Table 2, the exponential linear trend model and forecasting indicator analysis we conducted for the edX online program shows that there has been a significant increase in the cumulative number of online enrollment and learning across the HEI from the buildup of the pandemic (pre-Covid) through to the Covid-19 contingency period. The forecast was done using the (predictive) Forecast algorithm in Tableau (Tableau, 2020) to find the regular overall learning patterns in alignment to the measures that can be continued into the future. As shown in Fig. 4, not only does the result show that the peak of the online learning usage or record (approximately 4.2 million) was observed in the month of May, but it was forecasted that such increment in the learning margin or progression will remain equivalent or yet improve over the imminent months (post-Covid) of the pandemic.

**Table 2:** Exponential linear trend model and forecasting analysis

Trend Line Equation:	ln(Cumulative Count) = 0.00751102 * Month of Enrollment Period + Intercept					
Coefficients:	Value	StdErr	t-value	p-value		
Enrollment Period:	0.0075	0.00115	6.480	0.0029*		
Intercept:	-315.084	50.8909	-6.191	0.0034*		
Forcast Model formula:	Forecast indicator * ( Month of Enrollment Period + intercept)					
SSE (sum squared error):	0.086					
RMSE (mean squared error):	0.017					
R-Squared:	0.929					
Standard error:	0.131					
p-value (significance):	0.0025*					
Analysis of Variance:	Field	DF	SSE	MSE	F	p-value
	Forecast indicator	2	0.311	0.155	8.997	0.0220*

Significant level: *p* <  = 0.05

As represented in Fig. 4 and Table 2, the Exponential Linear trend model was computed for the natural log of sum of the Cumulative count (actual & forecast) given the Enrollment Period of 2020. The model which we implemented using the Tableau forecast tool (Tableau, 2020), iteratively predicts future values of each regular month from the weighted averages of the previous values of the chains or successions (Fig. 4). Typically, the method is said to be exponential because the value of each level of the estimated values (see: Fig. 4) is influenced by every preceding actual value to an exponentially decreasing degree, thus, more recent values are given greater weight. Consequently, as presented in Table 2, the quality of the Forecast model was determined through the Coefficient of Determination (R-squared) = 0.929, Root Mean Squared Error (RMSE) = 0.017, and Sum of Squared Estimate of Errors (SSE) = 0.086 (Le et al., 2020; Leitch & Tanner, 1991) which were acceptable, with significant value of *p* = 0.0025. Moreover, the Trend Line Equation (*p* = 0.0029) and Analysis of Variance of the model (*F* = 8.997, *p* = 0.0220) (Table 2 and Fig. 4) also shows to be significant.

#### Experiences and emotional well-being of teachers and students

**Fig. 5:**
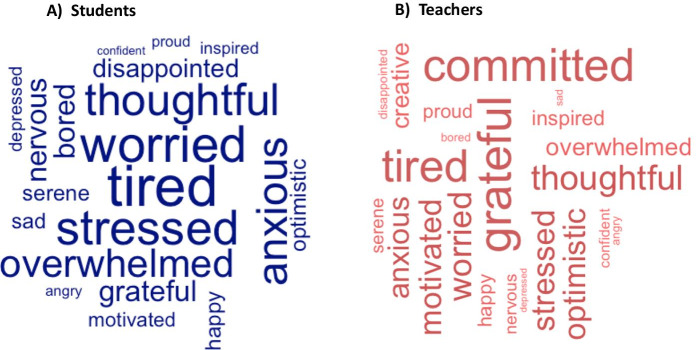
WordCloud representing the frequency of top emotional terms expressed by the students and teachers across the data. **a **Students, **b **Teachers

**Fig. 6:**
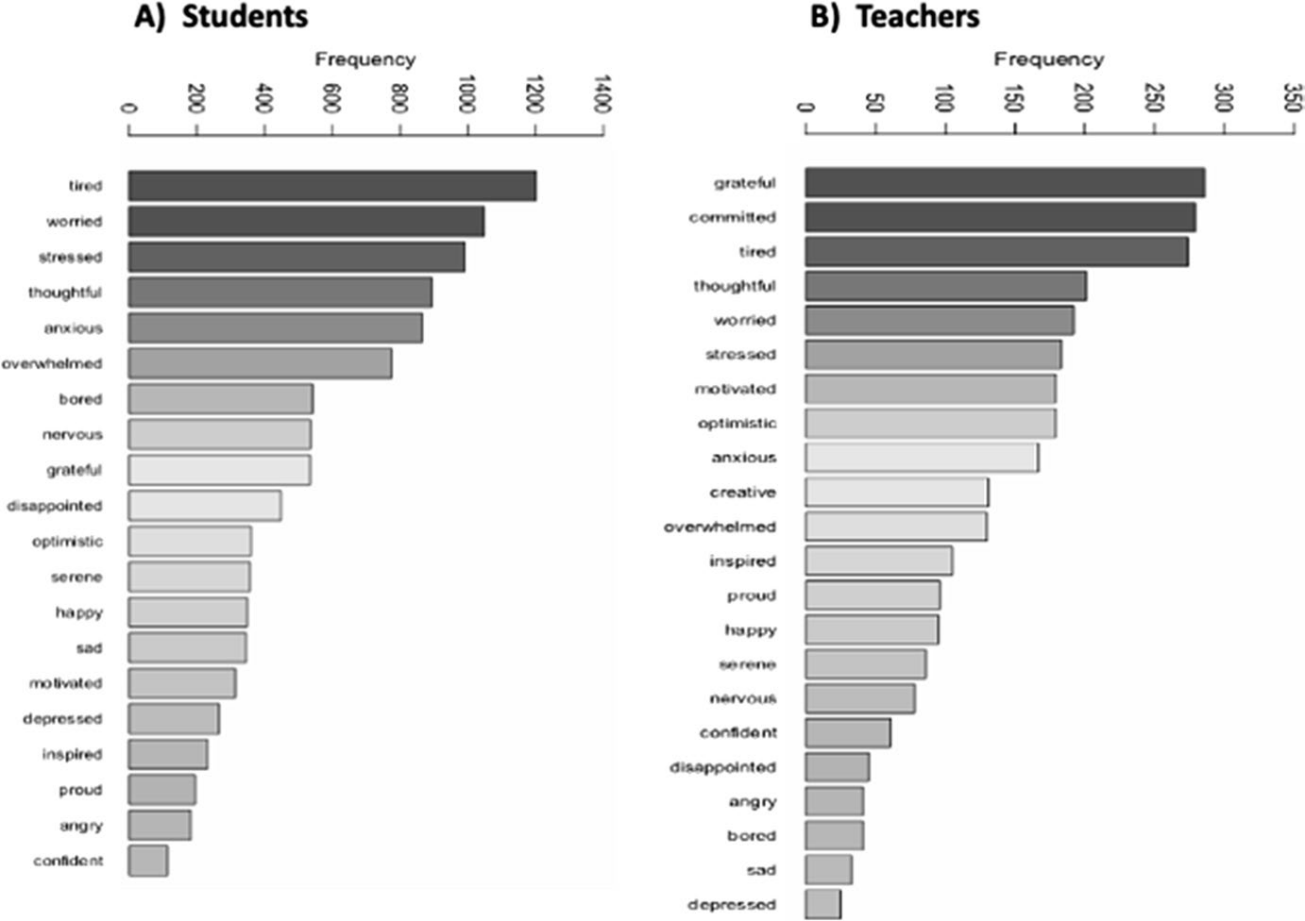
Chart showing the frequency of the top emotions by the students and their teachers. **a **Students, **b **Teachers

**Fig. 7:**
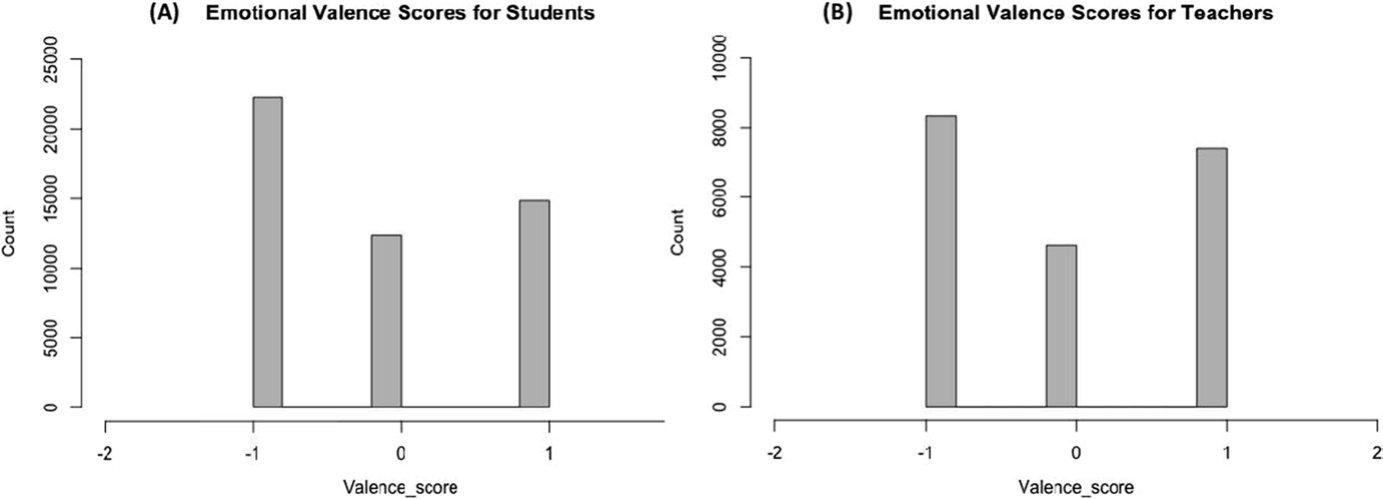
Emotional valence scores (polarization) for the students and teachers across the data. ** a **Emotional Valence Score for Students, **b **Emotional Valence Score for Teachers

**Fig. 8:**
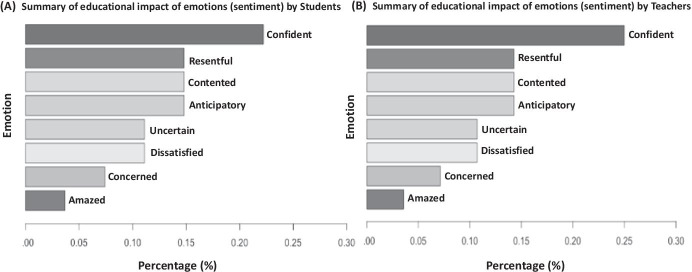
Chart representing the overall emotions (classification) expressed by participants across the data. **a **summary of educational impact of emotions (sentiment) by students **b **summary of educational impact of emotions (sentiment) by Teachers

**Fig. 9:**
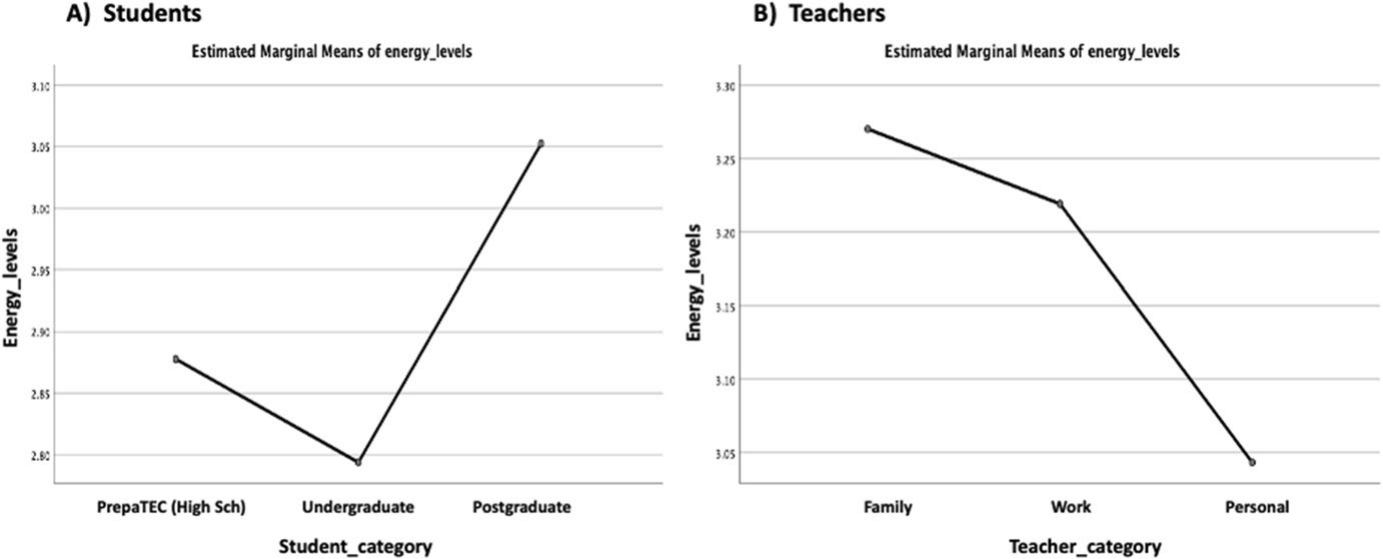
Energy levels broken down by students vs teachers category. **a **Students, **b **Teachers

**Fig. 10:**
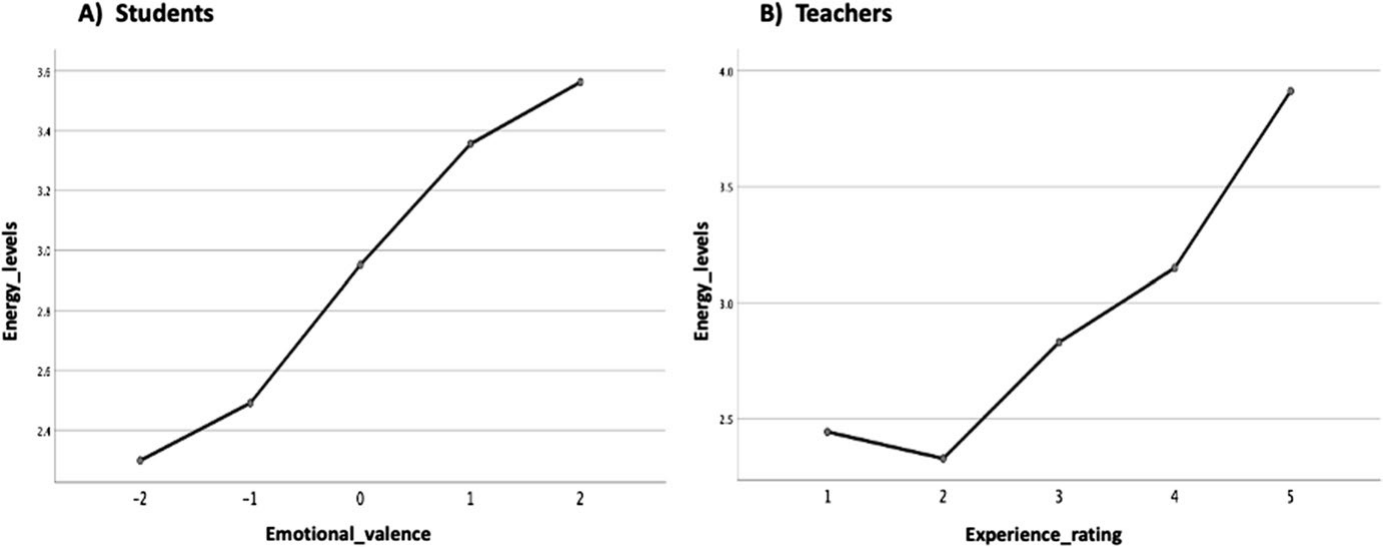
Energy levels broken down by students’ emotional valence and teachers experience rating. **a **Students, **b **Teachers

**Fig. 11:**
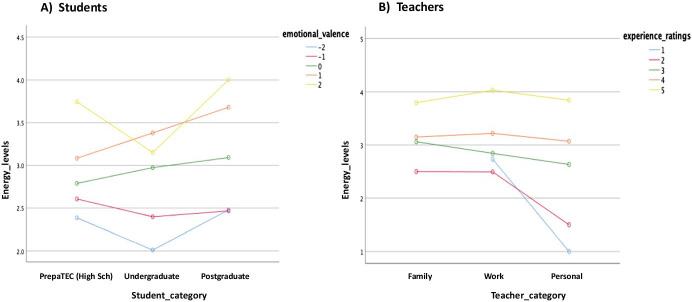
Energy levels plotted against emotional valence and experience rating considering the students vs teachers categories. **a **Students, **b **Teachers

**Fig. 12:**
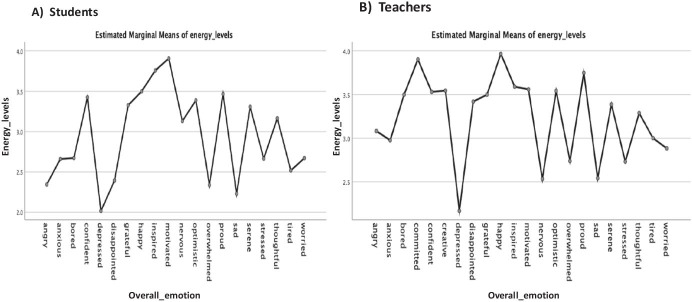
Energy levels plotted against the overall emotion broken down by students vs teachers. **a **Students, **b **Teachers

**Fig. 13:**
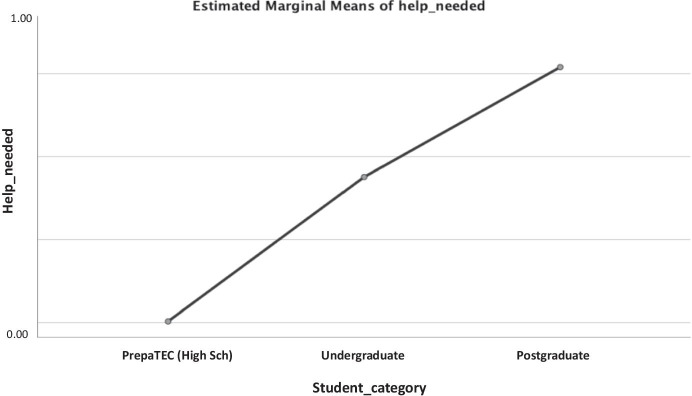
Marginal means of help_needed or not broken down by students category

**Fig. 14:**
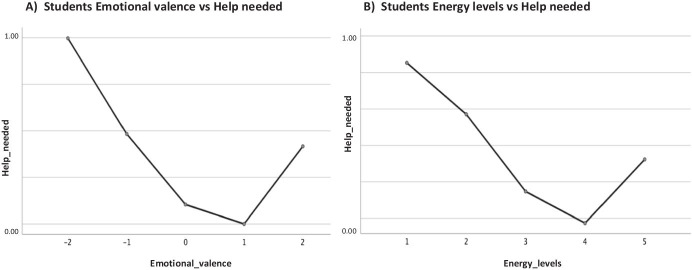
Marginal means of help_needed or not broken down by the emotional valence and energy levels of the students. **a **Students Emotional valence vs Help Needed, **b **Students Energy levels vs Help needed

**Fig. 15:**
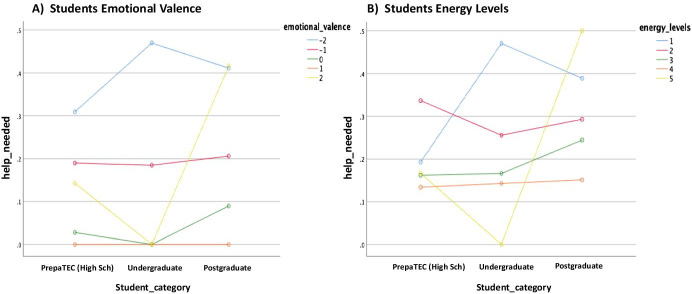
Help_needed plotted against emotional valence and energy levels of the students, respectively. **a **Students Emotional Valence, **b **Students energy levels

**Fig. 16:**
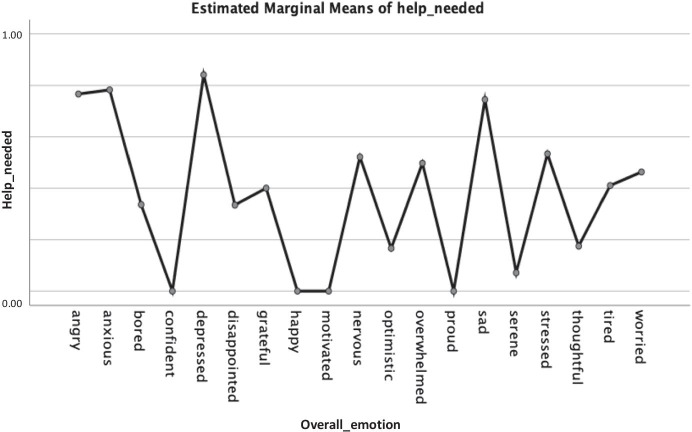
Estimated marginal means of help_needed or not broken down by overall emotions of the students

**Fig. 17:**
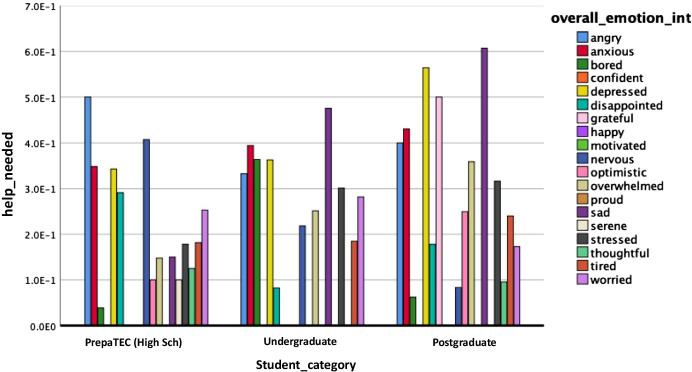
Help_needed vs overall emotions broken down by the students category

**Fig. 18:**
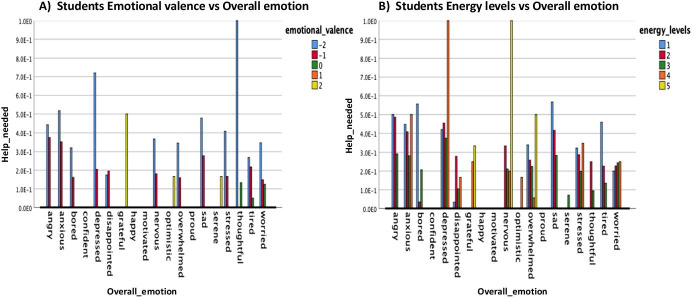
Help_needed vs overall emotions broken down by emotional valence and energy levels. **a **Students Emotional valence vs Overall emotion, **b **Students energy levels vs Overall emotion

Having predicted the learning progress of the students over the period of the Covid-19, we turn our attention to determining the extent to which the learning experiences and emotional well-being of the students and the teachers have impacted their learning and teaching during the Covid-19 contingency. To do this, first we analyzed the data from the MFD Emotions and Experience Questionnaire (TEC, 2020a) to determine the frequency and intensities (impact) of the emotional terms used or expressed by the students and their teachers across the data using R statistics tool (Rstudio, 2020), and then we conducted a univariate analysis of variance (ANOVA) using SPSS statistics (IBM, 2020) to determine the impact that the experiences, emotional valence, and the overall emotions have on the energy levels (learning motivation) taking into account the different categories of students and teachers. The results are as reported in the following Tables 3 and 4, and Figs. 5, 6, 7, 8, 9, 10, 11, 12, 13, 14, 15, 16, 17, 18).

**Table 3:** Effects that the students/teachers categories, overall emotions, experiences, and emotional valence have on the energy levels broken down by students vs teachers

Univariate tests of Between-Subjects Effects on the Energy levels for Students vs Teachers					
	Factor	Mean Sq	F	Sig	Partial Eta Sq
Students	student_category	3.409	6.873	0.001*	0.004
	emotional_valence	5.603	11.297	0.000*	0.012
	overall_emotion	2.730	5.505	0.000*	0.028
	student_category*emotional_valence	1.674	3.374	0.001*	0.007
	student_category*overall_emotion	0.667	1.344	0.078	0.014
	emotional_valence*overall_emotion	0.795	1.602	0.004*	0.023
	student_category*emotional_valence* overall_emotion	0.594	1.198	0.134	0.021
	overall contrast	4.688	9.453	0.000*	0.005
Teachers	teacher_category	1.896	4.096	0.017*	0.011
	experience_ratings	7.728	16.696	0.000*	0.080
	overall_emotion	1.311	2.833	0.000*	0.072
	teacher_category*overall_emotion	0.556	1.201	0.195	0.055
	teacher_category*experience_ratings	0.570	1.231	0.288	0.010
	overall_emotion*experience_ratings	0.768	1.658	0.004*	0.096
	teacher_category*overall_emotion* experience_ratings	0.583	1.260	0.144	0.056
	overall contrast	1.414	3.054	0.048	0.008

Sig. Levels *p* <  = 0.05, Energy_levels: Likert-scale 1–5

**Table 4:** Table showing the effects that the Students categories, energy levels, emotional valence, and overall emotions have on if they required help or not

Univariate test of Between-subjects Effects on Help_needed or not for the Students				
Factor	Mean Sq	F	Sig	Partial Eta Sq
student_category	0.036	0.194	0.824	0.000
energy_levels	0.005	0.025	0.999	0.000
emotional_valence	0.807	4.355	0.002*	0.008
overall_emotion	0.137	0.738	0.774	0.006
overall_emotion*student_category	0.109	0.586	0.959	0.008
overall_emotion*energy_levels	0.146	0.788	0.817	0.013
overall_emotion*emotional_valence	0.160	0.861	0.671	0.011
student_category*energy_levels	0.188	1.016	0.422	0.004
student_category*emotional_valence	0.170	0.920	0.490	0.003
energy_levels*emotional_valence	0.011	0.060	1.000	0.000
overall_emotion*student_category*energy_levels	0.169	0.912	0.651	0.021
overall_emotion*student_category*emotional_valence	0.158	0.850	0.682	0.010
overall_emotion*energy_levels *emotional_valence	0.164	0.886	0.638	0.011
student_category*energy_levels*emotional_valence	0.055	0.297	0.955	0.001
overall_emotion*student_category* energy_levels*emotional_valence	0.189	1.018	0.438	0.012
overall contrast	0.460	2.485	0.000	0.020

Sig. Levels *p* <  = 0.05, Help_needed scale: Yes = 1, No = 0

In Figs. 5 and 6, the work builds a corpus (library of words) of the top four emotions which students and teachers were asked to provide in addition to the main (overall) emotions they felt throughout the weeks. It is important to mention that the answers or reported emotions used for the text mining were in response to the following question “In addition to the (main) emotion you selected, what other emotions did you feel during this week?” which was a multiple-choice selection question of maximum of 4 emotions asked to the participants. The result of the text mining method using R statistics shows that whereas the students appears to be largely “tired”, “worried”, “stressed” and “thoughtful” (Figs. 5(a) and 6(a)). The teachers show to be mostly “grateful”, “committed”, “tired”, and “thoughtful (Figs. 5(b) and 6(b)). Perhaps, the aforementioned observations may be as a result of how appreciative the teachers were following the new elements of the hybrid learning model introduced to support the teaching–learning processes regardless of showing to be tired as a result of the Covid-19 outbreak/contingency plans.

Furthermore, we deemed it important to determine the impact (intensity levels) of the different reported emotions by the students and teachers in respect to the way they perceived or felt amidst the Covid-19 pandemic. To do this, we applied the emotional valence (sentiment analysis), a text mining method in R, which focuses on measuring (through polarization or terms quantification) the intensities of the different emotions reported by the stakeholder by extracting and assigning a (valence) score to each term (emotion) found. Technically, we used the *get_nrc_sentiment* function in R to extract the different (emotional valence) scores considering the responses by the students and their teachers. Typically, the get_nrc_sentiment functions by obtaining and quantifying (polarization) the intensities of the different terms (emotions) using the positive ( +), neutral (0) and negative (-) values (Litman & Forbes-Riley, 2004; Okoye et al., 2020) to represent each relevant term it finds in each case. In Fig. 7, we present the results of the method (valence scores) across the data considering the students and teachers. The values with positive valence ( +) scores represent an attractive emotion, whilst the negative (-) scores signify an aversive emotion. The zeros represent emotions that was classified as neutral (0) with no emotions or sentiment attached.

As gathered in Figs. 7 and 8, we note that, in overall, there were difference in the average margin of the different emotions reported by the teachers (min = -1, mean = -0.04, max = 1) (Fig. 7(b)) in comparison to the students (min = -1, mean = -0.15, max = 1) (Fig. 7(a)) valence scores. This was exclusively observed for the margin of positive or attractive emotions (+ 1) (Fig. 7). Interestingly, the aforementioned observation, can be triangulated or aligns with the findings we observed for the most frequently used terms (WordCloud) by the stakeholders (Figs. 5 and 6), in which we observed that the teachers were more positive than the students amidst the Covid-19 contingency plans and/or the implications we have explained earlier in Fig. 5 and 6. However, it can also be collectively said that a larger margin of the individual emotions reported by the students and teachers could be classified as aversive (-1) (Fig. 7).

The definition of *emotional valence* and its implication in respect to the different studied phenomenon or areas of its application, particularly within the educational domain, has been illustrated in the literature (Kort et al., 2001; Litman & Forbes-Riley, 2004; Okoye et al., 2020; Shen et al., 2009; Tian et al., 2010, 2018). As demonsttarted in this study, such type of analysis (allied to the text mining) is achieved by leveraging the underlying information (textual data) that are contained in the readily available datasets to draw useful insights or patterns about the population. Accordingly, as shown in Fig. 8, we adopted the emotion (sentiment) polarization or classifications of educational datasets as described in Litman and Forbes-Riley (2004) and Okoye et al. (2020) to classify the different types of emotions we have observed for the stakeholders. This was done in order to determine the overall implication of the reported emotions for the teaching–learning processes amidst the Covid-19 pandemic. It is noteworthy to mention, as reported in Fig. 8, that while the stakeholders’ trusts (confident) that the different contingency plans or learning platform/resources made available to them in the face of the pandemic may be effective to facilitate their various teaching–learning process. On the other hand, they were equally discontent (resentful), perhaps owing to the several challenges and implications of using the new learning platforms or methods for teaching–learning, which they may not usually have been accustomed to. Moreover, taking into account the teachers and students (Fig. 8), we found also that a greater proportion or percentage of the teachers’ population (approximately ~ 25%) indicated more trust towards the different measures or “new normal” than the students (< 25%).

Consequently, in order to understand the impact that the *overall emotions* and *experiences* of the students and their teachers have on the *energy levels* (teaching–learning motivation) factor or variable in the data (see: Table 1) by taking into account the significant differences and marginal mean effects; the study conducts a univariate analysis of variance (ANOVA) test. This was a test of between-subjects effects for the students vs teachers (energy levels) considering the students’ category, emotional valence, and overall emotion, and the teachers’ category, experience rating, and overall emotions (see: Table 1). It is important to mention for the analysis, the students’ categories were represented as interval values as follows: PrepaTEC (High Sch) = 1, Undergraduate = 2, and Postgraduate 3, and the teachers according to the main reason why they have expressed the different emotions as follows: Family = 1, Work = 2, and Personal = 3. The other variables we analyzed (overall emotions, experience rating, emotional valence, and energy levels) (see: Table 1), were all represented on a five-point ranked Likert-scale measurement with the emotional valence represented between -2 and 2 values to denote the intensities of the different emotions (i.e., negative (-), neutral (0) and positive ( +) values) (Litman & Forbes-Riley, 2004). The results of the univariate analysis for the students and teachers are as reported in Table 3 and Figs. 9, 10, 11, 12.

As reported in Table 3, we note that the students/teachers categories, emotional valence, experience ratings, and overall emotions differ and are all contributing factors to the energy levels (teaching–learning motivation) of the students and the teachers (*p* <  = 0.05) (see: Table 3). However, the test of between-subjects effect and comparison shows that while independently the targeted variables (student_category, teacher_category, emotional_valence, experience_ratings, overall_emotion) appear to be significant (*p* < 0.05), thus, influences the energy levels of the students/teachers. The students and their teachers did not take into account a combination of most of the different groups of variables, with only the student_category*emotional_valence (*F* = 3.374, *p* = 0.001) and emotional_valence*overall_emotion (*F* = 1.602, *p* = 0.004) appearing to be significant for the students, and overall_emotion*experience_ratings (*F* = 1.658, *p* = 0.004,) for the teachers, respectively. In other words, statistically, we can conclude that the energy levels or motivation for the students were also influenced by a combination of the “student_category”, “emotional_valence” and “overall_emotion” factors (student_category:emotional_valence, emotional_valence:overall_emotion) with a difference observed in terms of the students’ categories. While for the teachers, the energy levels was only influenced by combination of the “overall_emotion” and “experience_ratings” (overall_emotion:experience_ratings) regardless of the teachers’ category. In leu of the above findings, in Fig. 9, we note that the marginal means of energy level for the Postgraduate students were higher than the PrepaTEC (High Sch) and Undergraduate counterparts (Fig. 9(a)). Perhaps, this could be, purportedly, owing to the fact that the Postgraduate students are exposed to advanced and more rigorous learning settings, and besides, could be more adaptable to adverse circumstances such as the Covid-19 outbreak. However, it can also be said, independently, that the teachers who tend to consider more family-related feelings or emotion veer to have higher energy levels than when considering the work and personal interests (Fig. 9(b)). Moreover, the aforenoted observations may collectively reaffirm or align with the reason as to why the top four emotions we noted in Figs. 5 and 6, have correspondingly appeared for the students and teachers, vice-and-versa. Whereby, the teachers presented to be more appreciative (“grateful”, “committed”, “tired”, and “thoughtful”) of the new learning settings than the students (“tired”, “worried”, “stressed” and “thoughtful”) in the face of the pandemic or new normal.

Taking into account the emotional valence and experiences of the students and teachers, we found for both entities (Figs. 10 and 11) that the better (higher) the emotional valence and experiences are, the higher the energy level tends to be. Although, in Fig. 11, we note that the levels of emotional valence differ for the student categories in terms of the energy levels, with the highest rating (+ 2) appearing for the PrepaTEC and Postgraduates, and + 1 appearing for the Undergraduates (Fig. 11(a)).

In Fig. 12, we analyzed the marginal means of energy levels for the students and teachers by taking into account the overall reported emotions. We found that emotions such as “motivated” and “inspired” are contributing factors to the highest energy levels for the students, while “depressed”, “sad” and “overwhelmed” resulted in the lower energy levels (Fig. 12(a)). On the other hand, for the teachers, we note that “happy”, “committed” and “proud” resulted in higher energy levels, whilst, “depressed”, “nervous” and “sad” contributed to the lowest energy levels (Fig. 12(b)). Apparently, we could say from the results that the students and teachers who took on board the contingency plans and learning strategies that were put in place by the HEI developed a positive or higher energy level and attitude towards their respective teaching and learning process, while those who attached more of feelings and astound by the current Covid-19 circumstances and mode of learning tend to inadvertently display lower energy levels.

In that perspective, we turn our attention to students who have signified to feel some form of adverse emotion and were directed to answer the following question “You commented that you felt “main emotion” and that you find that emotion “unpleasant/very unpleasant”, do you consider that you need any help to be able to handle it?”. To do this, we took into account how their responses (i.e., needs help or not) differed by considering the students categories, energy levels, emotional valence, and overall emotion. It is noteworthy to mention that there were a total of *n* = 2475 (64%) participant that answered the question with 18% indicating they needed help and 46% indicating they do not. The results of the analysis are as presented in Table 4 and Figs. 13, 14, 15, 16, 17, 18).

As represented in Table 4, the only difference in students who indicated if they needed help or not was in terms of the emotional valence (*F* = 4.355, *p* = 0.002). Moreover, as shown in Fig. 13, the highest marginal means of help needed was observed for the Postgraduate students, whilst the Undergraduate students were borderline and PrepaTEC (High Sch) most likely did not seek for help or support. Also, when considering the emotional valence and energy levels of the students, we note in general that the students who have shown the lowest emotional valence (Fig. 14(A)) and energy levels (Fig. 14(B)) are the one who needed help most. When broken down by students’ category, we found that the most negative emotional valence (-2) (Fig. 15(A)) contributed to the highest proportion of students that needed help, except for the Postgraduate students who also took into account the positive emotions (+ 2) when doing so. The same pattern was observed for the students when considering the energy levels (Fig. 15(b)). While the lowest energy levels (i.e., 1 for Undergraduate, and 2 for PrepaTEC) contributed to the highest proportion of the students who needed help. On the other hand, a mixture of both (i.e., highest = 5, and lowest = 1) contributed to the proportion of Postgraduate students who needed help. Perhaps, we could say that this is owing to fact that the Postgraduate students are more open to seek help regardless of the circumstances as we noted earlier in the previous sections (Figs. 9 and 11).

Furthermore, in Figs. 16, 17, 18, we looked at the estimated marginal means of help needed or not considering the overall emotions of the students.

As shown in Fig. 16, we found that while emotions such as “depressed”, “anxious”, “angry” and “sad” contributed to the proportion of the students who needed the most help. Emotions such as “confident”, “motivated”, “happy” and “proud” resulted to the proportion of student that did not require any help with their learning process. When broken down by the students’ categories, we note again for the Postgraduate students that it was a mixture of both positive and negative emotions (sad, depressed, grateful) that have contributed to the students who needed help, as well as, the ones who did not need help (bored, nervous, thoughtful) (Fig. 17). The Undergraduate and PrepaTEC were slightly borderline in terms of students who needed help or not, with “sad” and “angry” being on top for the ones who needed help and “disappointed” and “bored” for the ones who did not need help, respectively (see: Fig. 17).

Last but not least, in Fig. 18, we look at the overall influence of the different emotions, energy levels and emotional valence expressed by the students in determining if they required help or not. We found that the combination of “thoughtfulness” and emotional valence of -2, being the lowest, contributed to the students who needed help, while “tired” and emotional valence neutral = 0, resulted in the ones who did not need help with their learning (Fig. 18(A)). On the other hand, we note that high energy levels (i.e., 4–5) and emotions such as “nervous” and “depressed” resulted in the highest proportion of students who needed help. Whereas, low energy levels (1–2) and emotions such as “bored” and “disappointed” also contributed to the ones who did not necessarily require help (Fig. 18(b)).

## Discussion

HEIs have invested huge resources and innovative strategies to provide technologies/mechanisms towards attaining effective teaching–learning experiences for the stakeholders under the current unprecedented time of Covid-19 pandemic. The response by the different educational institutions during this period has been remarkable, and will consequently change the way in which learning takes place even when the outbreak subsides (Woolliscroft, 2020). Perhaps, the lessons learned from the several contingency plans both in preparedness and aftermath of the pandemic, is not only to make sure that the HEIs do not replicate the failures of the pre-Covid era, but instead, build towards an improved educational system and acceleration of education and learning for all the stakeholders involved (e.g., teachers, students, education community, public, commercial, and industrial society, etc.) (Rogers & Shwetlena, 2020; UNESCO, 2014, 2015, 2016; UNICEF, 2018). The outcome of this study shows that with educational models such as the HyFlex + Tec model described in this paper, that the HEIs are not only capable of ensuring the continuity of education and learning progress for the learners. But also, are able to track and monitor the impacting factors, well-being, and experiences of the stakeholders (e.g., teachers and students) as demonstrated in the methodology of this paper.

Indeed, technology-mediated education have allowed the educators to continue business and academic services remotely in addition to ensuring that the teachers and students stay safe and healthy whilst learning during the pandemic (Chick et al., 2020; IEEE, 2020b; Reimers et al., 2020; Setiawan, 2020). However, given the fact that most of the educational curriculum has been designed to include face-to-face interaction especially for effective absorption of the delivered contents and experiences for the stakeholders. This study notes that any effort by the HEIs to implement and sustain a continuous education or programs as an aftermath of the Covid-19 pandemic must include the technological innovations that have spanned across this period. This ranges from ensuring that the students have an adequate digital technology literacy and infrastructures (IEEE, 2020a), to the effective delivery of online instructions and management of unforeseen clashes or events within the different learning platforms (Bao, 2020). Moreover, in the current wave and aftermath of the Covid-19, the rapid growth and enhanced access to information and communication technologies have never been more than ever affirmed to pose new possibilities towards teaching and learning in the diaspora (de Souza Rodrigues et al., 2020; Pettersson, 2020; Tzanavaris et al., 2020).

### Implications of this study

This study can be triangulated or allied to both pedagogical and technological factors and practices that may impact or influence the move to distance learning (technology-mediated education) amidst the Covid-19 pandemic. For instance, several educational technologies or innovations have been implemented by the HEIs with the primary goal of tracking, improving, and sustaining the students/staff wellbeing and teaching–learning process during the Covid-19 pandemic. However, whilst the findings of this study and the pieces of evidence we drew from the literature (see: Background information) (AIESAD, 2020; Bao, 2020; Chick et al., 2020; Kummitha, 2020; LALA, 2020; OECD, 2017; Reimers et al., 2020; UNESCO, 2020; Viner et al., 2020; Woolliscroft, 2020) purportedly shows that HEIs have invested in virtual technologies for teaching and learning purposes, and are becoming fast the next frontier in educational programs, e-content and curriculum development, following the aftermath of the Covid-19 pandemic (Almaiah et al., 2020). There still exists the portentous task of developing and implementing adequate and alternative solutions to fill the voids that those plans have and will consequently span. For instance, replacing the face-to-face (in-person) encounters or experiences for the stakeholders that supposedly constitutes a large part of the present-day educational models and curriculum (Armstrong-Mensah et al., 2020).

oreover, another implication of the technological advancements to the teaching and learning processes, as described in this study, will be to look into more pertinent teaching methods or platforms (be it in-person or virtual), along with development of (digital) teaching–learning skills for the stakeholders, to fully benefit from the technology-mediated education and process innovations (Almaiah et al., 2020; Mikheev et al., 2021; Mohan et al., 2020; Mpungose, 2020a; Raffaghelli et al., 2020). This means, alongside provision of the various promising learning platforms and programs, that HEIs must also focus their attention towards the digital literacy levels or training for the stakeholders to flourish in the modern day digital-savvy generation (Mpungose, 2020b; UNESCO 2014), as well as, how to fill the voids that the rapid shift from face-to-face to online learning has left for the stakeholders (Armstrong-Mensah et al., 2020). By so doing, the results of this study shows that there will also surface a *substantial* and *sustainable* increase in emotional well-being (comfortability-to-sustainability of learning) and attitude of the stakeholders towards the “new learning normal” (*digitalization*—otherwise allied to the technology-mediated education) amidst and in leu (aftermath) of the Covid-19 pandemic (Armstrong-Mensah et al., 2020; Burke, 2020; Di Pietro et al., 2020; Oyedotun, 2020; Pokhrel & Chhetri, 2021, UNESCO, 2014, 2016). For example, Di Pietro et al. (2020) attempted to gain a better understanding of how the COVID-19 pandemic may have directly or indirectly affected the students’ learning processes and outcomes. They note that students will suffer a learning loss, and that the effects will not affect students equally, and will influence both the cognitive and non-cognitive skills acquisition, including long-term consequences to short-term ones (Burke, 2020; Oyedotun, 2020). Interestingly, the study (Di Pietro et al., 2020) noted some elements that should be part of any successful strategies by the HEIs in integrating (online and offline) teaching–learning activities, to include; proper virtual learning environments (VLE), to guaranteeing access to internet and digital technologies for learning for the stakeholders, and teachers learning how to adapt their roles to situations in which they can effectively communicate with the students (e.g., to not lose motivation when shifting to the online learning platforms), through improved digital competences and pedagogical approaches (Mikheev et al., 2021; Raffaghelli et al., 2020) that are best suited for online learning and/or blended models such as the HyFlex + Tec model described in this study. To note, while Ma et al. (2021) found that teachers’ online teaching self-efficacy (TSE) in terms of technology-application for learning (Lin & Wang, 2021) increased during the Covid-19 lockdown. They (Ma et al., 2021) note that passion burnout (motivation) was a contributing factor towards the changes in online TSE for the teachers. Interesting, the aforenoted observation also aligns with the results of this study where we note that users’ emotions (positive or negative), and experiences for the teachers and students (see: Data Analysis and Results section) contributed to the energy levels (teaching-learning motivation), vice and versa.

Recent studies have also looked into how the transition to distance learning have impacted undergraduate vs graduate students, and how that information can be used to inform the university’s practices during crises such as the Covid-19 (Armstrong-Mensah et al., 2020). While the study of Armstrong-Mensah et al. (2020) notes that academic and technological needs of the students during the unprecedented time of the pandemic were exceptionally broad. They argued that it will be difficult to replicate the in-person learning experiences online, although majority of the students (69.9%) preferred asynchronous teaching style due to its flexibility, and the fact that they can learn at their own pace, at any time, and/or at any place. Besides, students who preferred synchronous style of teaching reported that it motivated and kept them up-to-date with learning, with more than half (53.6%) of the studied students’ population reporting that they were able to stay motivated and completed their learning activities on time amidst the Covid-19. Moreover, when considering the implication or long-term effect of the technology-mediated education for the HEIs, research has shown that remote learning can be as good or better than in-person learning for students who choose it (Burke, 2020). With the recent study by Pokhrel & Chhetri (2021) endorsing the different Covid-19 contingency plans by the HEIs as an opportunity to pave the way for ample adoption of digital education/learning, pointing out the most pressing need to be on how to “innovate” and “effectively implement” the alternative educational routines, learning management systems, or continuity strategies as described in this study.

## Conclusion

Technology-mediated education have become a fundamental part of modern-day teaching and learning following the aftermath of the Covid-19 pandemic. This study describes a hybrid learning model (HyFlex + Tec) that is used to support virtual and in-person learning processes for the stakeholders (teachers and students) within a higher educational institution. The study used the datasets from MOOCs and MFD Emotions Experience Survey from a University’ setting for its analysis. Theoretically, the study determined overall progress and statistics for the learners during the Covid-19 pandemic, as well as the impact that the teaching/learning experiences, emotional valence, and overall emotions expressed by the teachers and students have on their energy levels (motivation). This includes its influence on whether the students needed help or not during the Covid-19 learning setting. Overall, the study shows that technology-mediated education (e.g., the HyFlex + Tec model) ensured the continuity of education and learning for the stakeholders during the Covid-19 pandemic. It also proved useful in effective monitoring of the learning experiences and emotional well-being or feelings of the extended stakeholders (e.g., teachers, students, parents, educational community) following the aftermath of the pandemic (post-Covid) and the various learning settings and contigency plans put in place by the HEIs. Future works can adopt the hybrid model and methodology defined in this paper to understand and/or analyze datasets derived from the learning processes (information) about the learners within the different educational settings or context following the Covid-19 pandemic, or yet, reconstruction of the implemented method to include further types of analysis or components that may have not already been defined in this study.

## Data Availability

The datasets used and analyzed during this study are available from the corresponding author on request.

## Code availability (software application or custom code)

The codes used in performing the text mining analysis in this study are available from the corresponding author on request.

## Declarations

### Originality and review status

The authors confirm that this work is original and has not been published elsewhere, nor is it currently under consideration for publication elsewhere.
